# Validity of an algorithm for determining sleep/wake states using FS-760 in school-aged children

**DOI:** 10.1186/s40101-022-00303-2

**Published:** 2022-08-18

**Authors:** Minori Enomoto, Shingo Kitamura, Kyoko Nakazaki

**Affiliations:** 1 Department of Sleep-Wake Disorders, National Institute of Mental Health, National Center of Neurology and Psychiatry, 4-1-1, Ogawa-Higashi, Kodaira, Tokyo, 187-8553 Japan; 2 Department of Medical Technology, School of Health Sciences, Tokyo University of Technology, 5-23-22, Nishikamata, Ohta-ku, Tokyo, 144-8535 Japan

**Keywords:** Actigraphy, Polysomnography, Sleep/wake scoring algorithm, Validation, Children

## Abstract

**Background:**

Actigraphy is a method used for determining sleep (S)/wakefulness (W) by actigraph, a device equipped with a built-in accelerometer and an algorithm validated for each device. The S/W determination algorithm for the waist-worn actigraph FS-760 has been formulated for adults. However, the algorithm for children has not been established. The purpose of this study was to formulate an algorithm for discriminating S/W in school-aged children using FS-760 and to evaluate its validity. We further tested the generalizability of existing algorithm for adults by applying it to the children’s activity data and then examined factors associated with adult algorithm agreement rates by multiple regression analysis using combined adult and children data.

**Methods:**

Sixty-five, healthy, school-aged children (aged 6 to 15 years) were recruited and randomly assigned to two groups: A (*n* = 33) and B (*n* = 32). They underwent 8-h polysomnography (PSG) and wore FS-760 simultaneously to obtain activity data. To determine the central epoch of the sleep/wake states (𝑥), a five-order linear discriminant analysis was conducted using the activity intensity of group A for five epochs (𝑥_−2_, 𝑥_−1_, 𝑥, 𝑥_+1_, 𝑥_+2_; 10 min) and evaluate its accuracy with the activity of group B. To reveal the factors associated with adult algorithm agreement rate, we integrated the activity, age, sleep efficiency of 15 adults (aged 20 to 39 years) and those of 65 children for multiple regression analysis.

**Results:**

The mean agreement rate of the developed algorithm was 91.0%, with a mean sensitivity (true sleep detection rate) of 93.0% and a mean specificity (true wakefulness detection rate) of 63.9%. The agreement rate of the adult algorithm applied to children’s activity was significantly lower (81.8%) than that of the children algorithm. Multiple regression analysis showed that the agreement rates calculated by the adult algorithm were significantly related to mean activity of the 𝑥 epoch in NREM and REM sleep as well as age and sleep efficiency.

**Conclusions:**

The S/W states in school-aged children can be reliably assessed using the developed algorithm for waist-worn actigraph FS-760. Since the accuracy of the adult algorithms decreased when applied it to children which have different activity levels during sleep, the establishment and validation of population-specific S/W algorithms should be required.

**Supplementary Information:**

The online version contains supplementary material available at 10.1186/s40101-022-00303-2.

## Background

Polysomnography (PSG) is the gold-standard diagnostic method for evaluating sleep stages; it uses electroencephalography (EEG), electrooculography (EOG), and electromyography (EMG). While PSG can provide a detailed assessment of sleep status, it requires special equipment as well as skilled technicians, and it is hard to examine the long-term and continuous sleep status assessments. The primary sleep assessment method as an alternative to PSG is actigraphy. Actigraphy is a method using an actigraph, a small device equipped with a built-in accelerometer. By applying an activity intensity measured by actigraphs to the algorithm validated for each device [1–4], it is possible to determine sleep (S)/wakefulness (W). Although actigraphy cannot be used to assess detailed sleep stages, they are less invasive and do not interfere with the one’s living environment, thus making them suitable for screening and long-term sleep/wake recording under real-world situation objectively. In recent years, the use of actigraphy has expanded into various fields in public health studies [5–7].

As described, S/W algorithms of actigraphy were validated among various disorders and age groups including children [8–10], thus leading to the development of guidelines for diagnosing pediatric sleep disorders [11, 12].

The presence of these guidelines would cause the application of the algorithm only being validated with adults to children’s activity; however, the extrapolation is generally not recommended because of the lack of reliability. In fact, children tend to move a lot during sleep, which may underestimate the total sleep time measured by actigraphs [13]. Considering that children have different activity characteristics during sleep, it would be necessary to formulate a pediatric S/W scoring algorithm.

In addition to differences in overall nocturnal activity, children differ from adults in many aspects, so a multifactorial contribution is assumed if the accuracy of the algorithm is affected. Since children are characterized by weaker motor inhibition in REM sleep [14] and greater sleep need [15, 16], sleep stage-dependent activity, sleep efficiency, and other age-dependent factors may also be involved. It would also lead to accuracy issues in non-pediatric populations where such factors are common (e.g., patients with sleep-related breathing diseases with high activity during nighttime sleep). Therefore, clarifying what characteristics of children are relevant to sleep-wake determination algorithms will provide useful information for understanding methods for estimating sleep-wake from activity levels, as well as for applying the methods to various populations.

In this study, we evaluated the validity of waist-worn actigraph FS-760 (ACOS Co., LTD), which has only been validated with an adult, by formulating an S/W scoring algorithm for healthy, school-aged children. Among the sleep parameters to be estimated, we optimized sleep latency and wake after sleep onset time, as reported by Nakazaki et al. [4]. We also tested the generalizability of the existing algorithm for adults by applying it to the children’s activity data and then examined factors associated with adult algorithm agreement rates by multiple regression analysis using combined adult and children data.

## Results

### School-aged children S/W scoring algorithm

The S/W scoring algorithm was obtained by performing discriminant analysis using the activity intensity and PSG data (total 6900 epochs) obtained from the 33 participants in group A.

$$z=0.108294\ {x}_{-2}+0.147294\ {x}_{-1}+0.230126\ x+0.099353\ {x}_{+1}+0.059580\ {x}_{+2}$$

Here, *z* ≥ 1 denotes wake (W_ACT_) and *z* < 1 denotes sleep (S_ACT_). 𝑥_−2_, 𝑥_−1_, 𝑥, 𝑥_+1_, and 𝑥_+2_ indicate the activity intensity at 4 min before the evaluation epoch, at 2 min before the evaluation epoch, at 2 min after the evaluation epoch, and 4 min after, respectively.

### Validity of the school-aged children S/W scoring algorithm

The children algorithm formulated in this study was adapted to the activity data of group B, an independent group. The agreement rate, sensitivity, and specificity for each sleep stage were calculated (Table 1). The agreement rate for the entire recording period was 91.04 ± 4.94%, sensitivity 92.95 ± 6.32%, and specificity 63.88 ± 35.82%. In group A, the agreement rate was calculated in the same way. As the results showed no statistically significant differences between the two groups for any of the items, the following analyses were performed with the data by merging both groups.

**Table 1:** Accuracy of S/W determination using the FS-760 actigraph

		Group A	Group B	t	p
Agreement rate (%)	Overall	91.01 ± 5.41	91.04 ± 4.94	-0.02	0.984
	Stage 1	66.11 ± 30.29	57.35 ± 33.21	1.11	0.271
	Stage 2	92.27 ± 7.31	92.18 ± 7.73	0.049	0.961
	Stage 3+4	97.94 ± 2.32	98.29 ± 2.55	-0.584	0.561
	Stage REM	88.47 ± 19.59	93.09 ± 9.71	-1.210	0.232
	Stage W	66.99 ± 30.44	62.33 ± 33.42	0.587	0.560
Sensitivity (%)		93.17 ± 6.19	92.95 ± 6.32	0.138	0.891
Specificity (%)		66.85 ± 31.27	63.88 ± 35.82	0.356	0.723

### Optimizing the calculation of sleep parameters with the FS-760

**Fig. 1:**
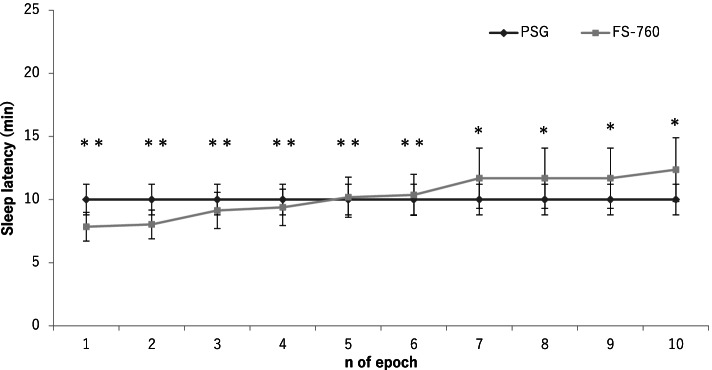
Optimization of sleep latency determined by the FS-760 (SL_ACT_). SL_ACT_ is the interval between the time lights were turned off and the time of the first S_ACT_ (sleep-onset time) among the sleep states that appeared continuously for more than *n* epoch for the first time after the lights were turned off. The horizontal axis shows the *n* defined above. The vertical axis shows sleep latency (min) defined for each *n*. *P < .01 and **P < .001, significant intraclass correlation between sleep latency determined by polysomnography (SL_PSG_) and SL_ACT_

**Fig. 2:**
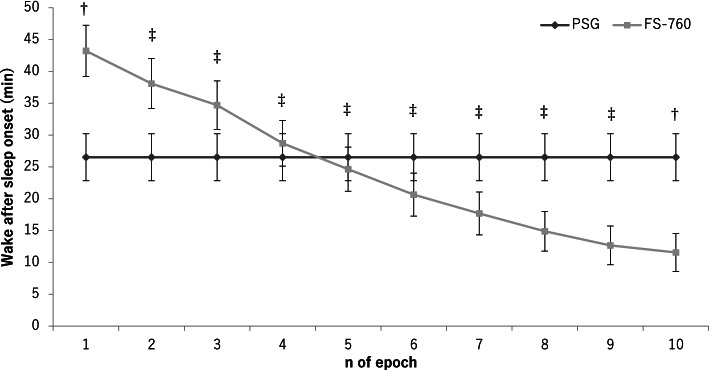
Optimization of wake after sleep onset determined by the FS-760 (WASO_ACT_). WASO_ACT_ is the total time that wake epochs determined by the FS-750 (W_ACT_) appeared continuously for more than *n* epochs after sleep onset. The horizontal axis shows the *n* defined above. The vertical axis shows wake after sleep onset (min) defined for each *n*. †*P* < .01 and ‡*P* < .001, significant difference between WASO_PSG_ and WASO_ACT_ (paired *t*-test). Values are expressed as mean ± SE

The number of consecutive S_ACT_ epochs in the definition of SL_ACT_ (see the “Methods” section) ranged 1–10 epochs showed no statistically significant difference between SL_ACT_ and SL_PSG_. When the number of epochs was 4 epochs, ICC was > 0.6 [17] and delta was minimum (Fig. 1). Therefore, the 4 consecutive S_ACT_ epoch sequences were adopted as the optimal condition for SL_ACT_. As in SL_ACT_, the ICC was > 0.6 and delta was minimal when the number of consecutive W_ACT_ epochs set at 5 for WASO_ACT_ (Fig. 2), we adopted it as the optimal condition for WASO_ACT_. The sleep parameters calculated by PSG and activity when optimal conditions (SL_ACT_: *n* = 4; WASO_ACT_: *n* = 5) were applied are shown in Table 2. There were no statistically significant difference between the sleep parameters calculated from the PSG data and those from the activity for any of the items; there was a significantly positive ICCs between the two parameters and improved after optimization for all the variables.

**Table 2:** Optimized sleep parameters determined by the FS-760 actigraph

	PSG	FS-760									
		Before optimization					After optimization				
	Mean±SE	Mean±SE	t	p	icc	p	Mean±SE	t	p	icc	p
Sleep latency (min)	10.00 ± 9.83	7.85 ± 9.16	-1.292	0.199	0.649	<0.001	9.38 ± 11.61	-0.326	0.745	0.654	<0.001
Wake after sleep onset (min)	26.52 ± 29.69	44.37 ± 34.27	3.173	0.002	0.491	0.001	24.65 ± 27.92	0.960	0.339	0.613	<0.001
Total sleep taime (min)	380.31 ± 32.24	364.62 ± 37.27	-2.567	0.011	0.524	<0.001	385.97 ± 34.96	-0.371	0.711	0.625	<0.001
Sleep efficiency (%)	90.55 ± 7.68	86.81 ± 8.87	-2.567	0.011	0.524	<0.001	91.9 ± 8.32	0.960	0.339	0.613	<0.001

### Accuracy of adult S/W algorithms in school-aged children

Table 3 shows the agreement rate, sensitivity, and specificity for each sleep stage of the S/W scoring, as calculated by adapting adult S/W scoring algorithms to the activity data of 65 children. The accuracy of the adult S/W scoring algorithm was significantly lower than that of the developed algorithm with children, except for the rate of agreement and specificity for Stage W (agreement rate, sensitivity, and specificity are 81.84 ± 8.83%, 81.64 ± 10.62%, and 79.17 ± 28.47%, respectively). On the other hand, the result of adapting the adult scoring algorithm to the activity data of the 15 adults showed that the accuracy was almost the same as that of existing reports [4, 18]: agreement rate 84.41 ± 1.88%, sensitivity 91.15 ± 1.91%, and specificity 53.19 ± 3.96%.

**Table 3:** Comparison of accuracy between school-aged children and adult S/W algorithms adapted to children's data respectively

		Algorithm for child	Algorithm for adult	t	p
Agreement rate (%)	Overall	91.03 ± 5.14	81.84 ± 8.83	-7.254	<0.001
	Stage 1	61.8 ± 31.82	37.12 ± 31.23	-4.462	<0.001
	Stage 2	92.23 ± 7.46	78.28 ± 12.78	-7.600	<0.001
	Stage 3+4	98.11 ± 2.43	93.18 ± 6.08	-6.085	<0.001
	Stage REM	90.74 ± 15.59	76.07 ± 22.03	-4.383	<0.001
	Stage W	64.70 ± 31.78	77.11 ± 27.27	2.390	0.018
Sensitivity (%)		93.06 ± 6.20	81.64 ± 10.62	-7.490	<0.001
Specificity (%)		65.39 ± 33.36	79.17 ± 28.47	2.533	0.013

### Differences in activity intensity between adults and children

Two-way analysis of variance showed no significant interaction between the group and epochs. However, there was a significant main effect of the group; activity intensity was significantly higher in children than in adults during all sleep stages (W, NREM, and REM) (see Supplemental Fig.).

### Searching for factors contributing to the accuracy of the adult S/W algorithm

To clarify the factors related to the agreement rate, we conducted a multiple regression analysis using the agreement rate of the adult algorithm as the objective variable (Table 4). Since the multicollinearity was not violated, all five explanatory variables (age, sleep efficiency, and the average activity of each 𝑥 during Wake, NREM sleep, and REM sleep) were included. Adjusted-*R*^2^ (0.720) indicated that the model was judged to be a good fit (F (5,70) = 39.602, *P* < 0.0001). Agreement rates calculated by the adult algorithm were significantly associated with the mean activity in NREM and REM sleep of 𝑥 epoch as well as age and sleep efficiency. On the other hand, the average activity of 𝑥 epochs of W showed no significant association.

**Table 4:** Multiple regression analysis with agreement rate of algorithm for adults as the objective variable

	β	t	p
age	-0.122	-1.778	0.080
Average activity of REM in x epoch	0.137	2.204	0.031
Average activity of NREM in x epoch	-0.903	-10.898	<0.0001
Average activity of Wake in x epoch	-0.016	-0.195	0.846
sleep efficiency	0.386	5.929	<0.0001
R^2^	0.720***		

β partial regression coefficient, ***P < 0.0001

## Discussion

In this study, we developed an S/W scoring algorithm for the FS-760 in healthy school-aged children.

First, we established the five-order linear discriminant equation as the S/W scoring algorithm using a total of five epochs of activity, including two epochs before and after, following previous studies using group A’s activity data and PSG’s sleep scoring data. The formulated S/W scoring algorithm was applied to the activity data of group B, which was an independent population with no difference in age, sex, or body size, to determine S/W. The results showed that the agreement with PSG data was 90.92 %. Out result was comparable to the agreement rates which have been reported in the range of 85 to 96% [1–4, 19–22]. The sensitivity and specificity also showed a trend of high sensitivity and low specificity, as in many previous reports [1–4, 20, 21].

One of the problems with the use of actigraphs has been pointed out the difficulty in detecting wake [23, 24]. In particular, it was difficult for actigraphy to distinguish silent awakeness from sleep based on the activity [1, 21, 25, 26]. When using the algorithm developed in this study, the specificity with PSG data was approximately 63%, which is a little lower than that reported for the FS-760 adult algorithm (65%) [4] but approximately the similar or higher than in the other previous reports (34–58%) [3, 21, 27]. In this study, optimization of the definition of sleep parameters was conducted following previous studies; the optimal settings were four consecutive epochs for sleep onset latency and five consecutive epochs for wake after sleep onset. When the sleep variables were recalculated by adapting this optimization, there was no longer a significant difference in those calculated by PSG in all sleep variables (SL, WASO, TST, and SE). Thus, the sleep variables calculated using this algorithm were reasonable. Note that the data used in this study were obtained from the first night PSG, so it is likely that the first night effect [28] is occurring in WASO.

To examine the generalizability of the adult S/W scoring algorithm, we applied it to the activity data of 65 school-aged children in the present study; then, S/W scoring was conducted. The overall agreement rate of the adult S/W algorithm was significantly lower than that of the school-aged children. Although the accuracy of specificity increased by approximately 12 points, the accuracy of the other parameters decreased by approximately 10 points.

To clarify the factors associated with this decreased inaccuracy, multiple regression analysis was conducted using the agreement rate of adult algorithm as the objective variable with integrated the 80 data of adults and children. Results showed that the average amount of activity during sleep, sleep efficiency, and age were significantly associated. Since children were more active during sleep, their S/W determination algorithm must use a high threshold to determine W. In fact, the coefficients of their S/W scoring algorithm was smaller than those of the adult algorithm. When the adult S/W scoring algorithm with the lower threshold was applied to the children’s activity data, the probability of being scored as W, with a discrimination score *z* ≥ 1, further increases. It has been reported that children shifted their body position during sleep more frequently than adults [13], and that the accuracy of S/W scored by the actigraph was lower [8, 10]. Considering that there are differences in the amount of activity during sleep between children and adults, and that the activity during sleep was also a major determinant of accuracy during the multiple regression analysis, the population with higher activity levels during sleep due to concomitant sleep disorders including sleep apnea, and inappropriate sleep environment (noise, inappropriate temperature/humidity/bedding, etc.) were highly likely to show the similar inaccuracy. For the population, it was necessary to validate a unique S/W determination algorithm that considers population activity characteristics. A low accuracy has been reported in such populations [24, 29]. As revealed by the multiple regression analysis in this study, age and sleep efficiency may have contributed to the accuracy, which were independent of activity. These results were consistent with existing studies [30].

Although the results of this study indicated the need to validate a unique S/W determination algorithm for children, there were reports showing that the accuracy of the adult algorithm may be maintained even when adapted to children. In these reports, the agreement rate with PSG were 87–90% [9, 29, 31, 32]. One possible factor causing difference between these reports and the present study is the site of attachment of the actigraph. The previous reports attached actigraphs to the non-dominant wrist (extremity region) while we attached it the waist (lumbar region) in the present study. Differences in the activity intensity depending on the attached site have also been reported. Previous reports have shown lower activity in the trunk than in the wrist [33]. Position shifts during sleep were more common in children than in adults [13]; since the actigraph FS-760 measured the activity of the trunk, the amount of body movements during sleep, which is usually small in adults compared to the peripheral, is relatively large in children. This may account for the discrepancy with the results of the previous studies. On the contrary, Paavonen et al. reported that in children aged 7–12 years, the actigraph is equally accurate at the wrist and the waist [34], suggesting the discrepancy may depend on the device characteristics.

As mentioned above, the amount of activity may differ between the wrist and trunk, and the accuracy of the actigraph may vary. However, the results of this study indicate that a waist-worn actigraph has sufficient accuracy to determine the S/W as well as a wrist-worn actigraph. Wearing the actigraph on the waist is less burdensome and less conspicuous compared to the wrist. Therefore, waist-worn actigraph is considered more applicable to children who are not accustomed to wearing wristwatches.

This study has several limitations: the children included in this study were 6–15 years old; only elementary to middle-school students were included in the study. Therefore, preschoolers, including newborns, should also be considered in future studies. In addition to age, only healthy subjects without sleep disturbances were included in this study. Because the actigraph used in this study was worn on the trunk, it may be necessary to examine the differences in the wearing site in future studies. FS-760 is a simple wearable device that can measure only activity intensity. In the future, it is one of the possibilities to consider further enhancing its accuracy as a multi-wearable device by combining it with a heart rate monitor and other devices [35]. The time epoch for the FS-760 used in this study is 2 min; 4 consecutively scored sleep stages (1 epoch = 30 s) were re-classified as either sleep (sleep epochs determined by PSG, S_PSG_) or wake (wake epochs determined by PSG, W_PSG_) every 2 min. So it is possible that Wpsg is more likely to contain obvious wakes rather than W in PSG, and that this procedure may lead the high specificity.

## Conclusion

In this study, we developed an algorithm for the S/W scoring of a waist-worn actigraph FS-760 in school-aged children. The resultant algorithm was then validated using PSG data to determine whether its accuracy in children was similar to that in the adults. We also examined the differences between the resultant and established adult algorithms and clarified the importance of validating the scoring algorithm for specific population. Our results may be applied in the fields of sleep re-exploration and sleep medicine in the future, particularly in interventions targeted for school-aged children.

## Methods

### Formulation of the algorithm for school-aged S/W scoring

#### Subjects

Sixty-seven school-aged children participated in this study. Screening PSG, questionnaires and medical examination of children and their caregivers confirmed that the participants had no severe mental, physical, or sleep disorders. Two children who were unable to complete the protocol were excluded from the analyses. Finally, we recruited 65 children (41 boys, 24 girls; mean age 10.5 ± 2.6 years, aged 6 to 15 years) for this study. Following the split-sample method [36], they were randomly assigned to two groups: group A (33 participants) and group B (32 participants). There were no significant differences in %male, age, height, weight, and BMI between groups (Table 5).

**Table 5:** Demographic data

	Group A	Group B	Whole	t/χ2	p
n	33	32	65		
%male	60.6	65.6	63.1	0.026	0.871
Age (y)	10.5 ± 2.7	10.4 ± 2.6	10.4 ± 2.6	0.215	0.831
Height (cm)	143.2 ± 15.8	141.5 ± 15.6	142.4 ± 15.6	0.446	0.657
Weight (kg)	38.4 ± 11.7	37.1 ± 13.3	37.7 ± 12.4	0.415	0.679
BMI (kg/m2)	18.3 ± 2.7	17.9 ± 2.8	18.1 ± 2.7	0.602	0.55

#### Procedure

This study was conducted in the sleep laboratory unit of the National Institute of Mental Health, National Center of Neurology and Psychiatry. We simultaneously recorded the sleep state and activity intensities during sleep using PSG and actigraphy.

#### PSG recording

The lights-out time in the laboratory was determined according to the participants’ habitual bedtime in their home records (sleep diary) preceding the experiments. In principle, the time in bed was set at 7 h; subjects were instructed to not get up if they woke up in the middle of the night and to get as much sleep as possible until lights-on. For those whose bedtime exceeded 7 h, data from the first 7 h were used for analysis; for those whose bedtime was less than 7 h, all data were used for analysis. The unit was maintained at 25°C and 50% relative humidity (RH). PSG recordings were made using Neurofax digital EEG system (EEG-1200, Nihon Kohden, Tokyo, Japan), which included an EEG with a conventional montage (F_3_, F_4_, C_3_, C_4_, O_1_, O_2_) based on the contralateral mastoid (M_1_, M_2_), an EOG at the outer canthus of each eye, a submental EMG, and an electrocardiogram (ECG). Upon recording, the EEG, EOG, EMG, and ECG signals were digitized at 200 Hz; the signal was filtered using a high-pass filter with the following time constants: EEG 0.3 s, EOG 0.03 s, submental EMG 0.03 s, and ECG 1.0 s. The signal was filtered using a low-pass filter as following: EEG 60 Hz, EOG 60 Hz, submental EMG 60 Hz, and ECG 60 Hz. The sleep stage (Stage N1, Stage N2, Stage N3, Stage R, or Stage W) was determined every 30 s according to the American Academy of Sleep Medicine (AASM) Manual for the scoring of sleep and associated events [37]. Four consecutively scored sleep stages (1 epoch = 30 s) were re-classified as either sleep (sleep epochs determined by PSG, S_PSG_) or wake (wake epochs determined by PSG, W_PSG_) every 2 min; they corresponded with the activity intensity data measured by the FS-760 (1 epoch = 2 min). When four consecutive data contained two or more Stage W parameters, the dataset was classified as wake (W_PSG_) according to the definition adopted by previous studies [2–4, 38]. On the other hand, all other datasets were classified as sleep datasets (S_PSG_). Furthermore, S_PSG_ was sub-classified as Stage R, Stage N1, Stage N2, or Stage N3, according to the most frequent sleep stage in the epoch (e.g., when S_PSG_ contained three or more Stage N1 data, it was classified as Stage N1). However, when S_PSG_ contained two different stages, the priority order (Stage R → Stage N1 → Stage N2 → Stage N3) was used (e.g., when S_PSG_ contained two stages N1 and R, it was classified as Stage R).

### Activity recording with the FS-760

Activity during the night was recorded using the waist-worn FS-760 (ACOS CO., LTD, Nagano, Japan). This small, rectangular device had the same accelerometer as that in the FS-750, which was validated by Nakazaki et al. for adults [4].

Briefly, the FS-760 is equipped with a three-axis accelerometer, which count the number of times the acceleration exceeds a reference value every 0.125 s and sums it at 2-min intervals. From this record, 32 levels of activity intensity are calculated and stored.

### Formulation of the algorithm for S/W scoring

To develop an algorithm for the FS-760 that determines the S/W states, a five-dimensional linear model was adopted according to previous studies [3, 4]. We hypothesized that this model utilizes activity intensity at an evaluation epoch as well as two epochs before and two epochs after (total of 10 min). Using the activity intensity at 4 and 2 min before the evaluation epoch, at the evaluation epoch, and at 2 and 4 min after the epoch (𝑥_−2_, 𝑥_−1_, 𝑥, 𝑥_+1_, 𝑥_+2_), each with a weighting coefficient (𝑎_−2_, 𝑎_−1_, 𝑎, 𝑎_+1_, 𝑎_+2_), the following equation gives composite variable *z*, which is the discriminant score:

$${z= a}_{-2}{x}_{-2}+{a}_{-1}{x}_{-1}+\mathrm{a}x+{a}_{+1}{x}_{+1}+{a}_{+2}{x}_{+2}$$

The criteria for the linear discriminant equation were S_PSG_ (= 0) and W_PSG_ (= 1) obtained with PSG (1 epoch = 2 min). The coefficients for classifying the activity intensity obtained from the FS-760 into sleep (S_ACT_) and wakefulness (W_ACT_) according to the above formula were obtained by linear discriminant analysis using a data set containing activity intensity and PSG data from 33 subjects in Group A. We adopt the split-sample method for this study [36].

### Validity of the school-aged children S/W scoring algorithm

Using the newly developed S/W scoring algorithm, the overall agreement rate, sensitivity, and specificity were calculated for the entire recording period as well as for each sleep stage (Stage N1, Stage N2, Stage N3, Stage R, or Stage W) for each participant in group B. These parameters indicated how sleep scores by PSG (S_PSG_, W_PSG_) closely match the estimates by activity intensity (S_ACT_, W_ACT_) for each corresponding epoch. Sensitivity was defined as the ratio of S_ACT_ to S_PSG_ during the entire recording period. Specificity was defined as the ratio of W_ACT_ to W_PSG_ during the entire recording period. The agreement rate for each sleep stage determined by PSG (Stage N1, Stage N2, Stage N3, Stage R, or Stage W) was defined as the percentage of activity intensity scores (S_ACT_ or W_ACT_) that closely match those calculated for each sleep stage. Similarly, the agreement rate in group A was calculated; it was considered whether there was any difference from the agreement rate in group B.

### Optimization of the definition of sleep parameters

Sleep latency (SL), total sleep time (TST), wake after sleep onset (WASO), and sleep efficiency (SE) were calculated using the S/W data obtained from the PSG and activity intensity data for each 2-min epoch [37].

The definitions of sleep parameters calculated from the PSG data are as follows: (1) SL_PSG_: the interval between the time of lights-off and the time of the first epoch when any of the sleep stages appeared (sleep-onset time); (2) TST_PSG_: the total time period when sleep (S_PSG_) appeared from the time of sleep onset to the time of lights-on; (3) WASO_PSG_: time of (Time in bed (TIB) – (SL_PSG_ + TST_PSG_); and (4) SE_PSG_: the ratio of TST_PSG_ to TIB.

The definitions of sleep parameters calculated from the activity intensity data are as follows: (1) SL_ACT_: the interval between the time of lights-off and the time of the first S_ACT_ (sleep-onset time) among the sleep states that appeared continuously for more than *n* epochs for the first time after the time of lights-off, where *n* ranged from 1 to 10 (2 to 20 min) and the SL_ACT_ was calculated for each occurrence; (2) WASO_ACT_: the total time of W_ACT_ that appeared continuously for more than *n* epochs after (optimized) sleep onset, where *n* ranged from 1 to 10 (2 to 20 min) and the WASO_ACT_ was calculated for each occurrence. When W_ACT_ appeared continuously for more than *n* epochs, the epochs were defined as W_ACT_; (3) TST_ACT_: TIB from which SL_ACT_ and WASO_ACT_ were subtracted; and (4) SE_ACT_: the ratio of TST to TIB.

For the calculation of SL_ACT_ and WASO_ACT_, the values obtained from the criteria applied above to the values of SL_PSG_ and WASO_PSG_ were compared; the epoch numbers that would optimize the calculated results were sought. The optimization rules were to minimize the difference between the average parameter values obtained by PSG for the 65 participants and the average parameter values obtained from the S/W algorithm, such that the difference was not significant. The intraclass correlation coefficient (ICC) was considered sufficient when the ICC was ≥ 0.6 [17].

### Accuracy of adult S/W algorithms in children

To confirm whether the accuracy of the existing algorithm for FS760, which was established with adults, can be used for children, the algorithm of Nakazaki et al. [4] was adapted to the activity data of our subjects (65 children). After adaptation, we calculated the agreement rate, sensitivity, and specificity of PSG S/W scoring for the entire recording period and each sleep stage (Stage W, Stage N1, Stage N2, Stage N3, and Stage R).

### Differences in activity intensity between adults and children

As a background factor for using different algorithms for children and adults, we compared differences in the amount of activity during sleep between children and adults. Differences in activity intensity between adults and children according to sleep stage (Wake, NREM, and REM) were compared.

### Searching for factors contributing to the accuracy of the adult S/W algorithm

To identify the factors associated with adult S/W scoring agreement rates in the activity data, we used concurrently recorded PSG and activity data from 17 adults who participated in the other studies as well as those from 65 children of this study. Of the 17 adults, one with an apnea hypopnea index (AHI) > 5 and another with periodic limb movement index (PLMI) > 15 were excluded; finally, 15 adults (14 males and 1 female, mean age 26.8 ± 6.0 years, aged 20 to 39 years) were included in the analysis. Activity data of adults were acquired using FS-760 or compatible actigraph MTN-220 (ACOS CO., LTD) [39] and processed in the same way as for the children. The adult S/W scoring algorithm was applied to the activity data of 80 people; the agreement rate was then calculated. Multiple linear regression was conducted to reveal the factors associated with the agreement rates using the adult S/W scoring algorithm with age, sleep efficiency, and the average activity of each 𝑥 when 𝑥 was Wake, NREM sleep, and REM sleep.

### Statistics

Unpaired *t*-tests were used to compare the sensitivity, specificity, and agreement rates between groups A and B for both the entire recording period and each sleep stage. Paired *t*-tests and ICC were performed to compare sleep parameters determined from PSG and activity intensity data obtained before and after the application of the optimization rules. Two-way analysis of variance with group (adult/ children)✕ epoch (𝑥_−2_, 𝑥_−1_, 𝑥, 𝑥_+1_, 𝑥_+2_) was used for the comparison of differences in activity intensity between adults and children. Multiple linear regression analysis was performed using the forced entry method. All data are expressed as the mean ± SD. All statistical analyses were performed using R (version 3.6.1; R Foundation) and IBM SPSS Statistics (version 26.0; IBM). Statistical significance was set at *P* < 0.05.

## Supplementary Information

**Additional file 1.** A description of this figure is provided in the text.

## Data Availability

The datasets during and/or analyzed during the current study are available from the corresponding author on reasonable request.

## Abbreviations

AHI: Apnea and hypopnea index
ECG: Electrocardiogram
EEG: Electroencephalogram
EMG: Electromyogram
EOG: Electrooculogram
ICC: Intraclass correlation coefficient
PLMI: Periodic limb movement index
PSG: Polysomnography
RH: Relative humidity
S_ACT_: Sleep epochs determined by the FS-760
SE: Sleep efficiency
SL: Sleep latency
SL_ACT_: Sleep latency determined by the FS-760
SL_PSG_: Sleep latency determined by PSG
S_PSG_: Sleep epochs determined by PSG
S/W: Sleep/wake
TIB: Time in bed
TST: Total sleep time
W_ACT_: Wake epochs determined by the FS-760
WASO: Wake after sleep onset
WASO_ACT_: Wake after sleep onset determined by the FS-760
WASO_PSG_: Wake after sleep onset determined by PSG
W_PSG_: Wake epochs determined by PSG
