# Supplementary material for: Validity of an algorithm for determining sleep/wake states using FS-760 in school-aged children
Source: J Physiol Anthropol. 2022 Aug 18;41:29. doi: 10.1186/s40101-022-00303-2 (PMC9387041; doi:10.1186/s40101-022-00303-2)
Supplement: Supplementary file 1 — Additional file 1. A description of this figure is provided in the text. [file 40101_2022_303_MOESM1_ESM.pptx]

## Slide 1
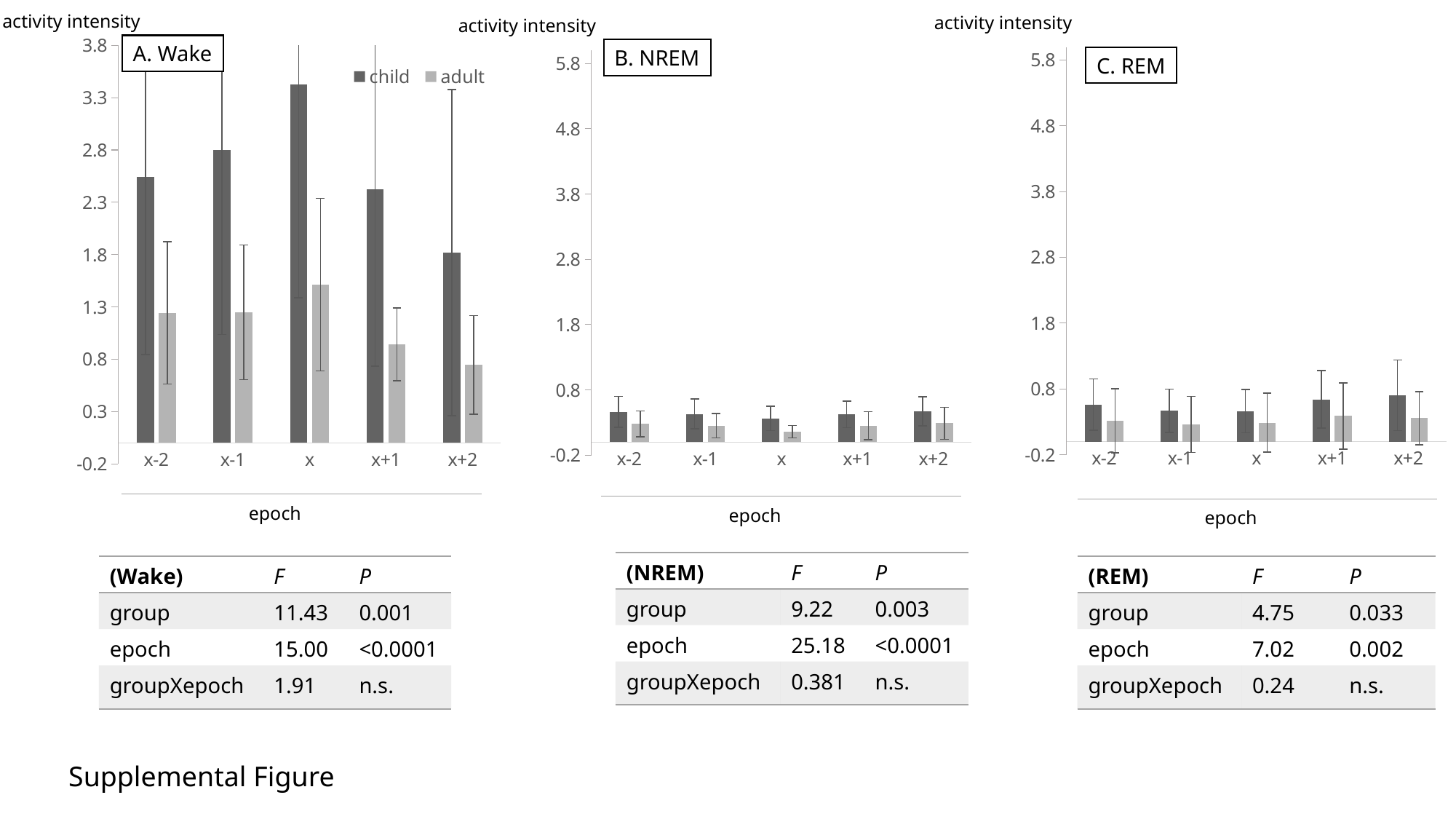

activity intensity
activity intensity
activity intensity
### Chart
| Category | child | adult |
|---|---|---|
| x-2 | 2.539891 | 1.241961 |
| x-1 | 2.800456 | 1.247334 |
| x | 3.427247 | 1.513627 |
| x+1 | 2.424654 | 0.941775 |
| x+2 | 1.818197 | 0.744706 |
### Chart
| Category | child | adult |
|---|---|---|
| x-2 | 0.561785 | 0.31643 |
| x-1 | 0.468391 | 0.259382 |
| x | 0.463427 | 0.28635 |
| x+1 | 0.642167 | 0.388467 |
| x+2 | 0.702017 | 0.354433 |A. Wake
### Chart
| Category | child | adult |
|---|---|---|
| x-2 | 0.462661 | 0.282475 |
| x-1 | 0.432002 | 0.253163 |
| x | 0.363888 | 0.159712 |
| x+1 | 0.42491 | 0.25029 |
| x+2 | 0.472536 | 0.289617 |B. NREM
C. REM
epoch
epoch
epoch
| (NREM) | F | P |
| --- | --- | --- |
| group | 9.22 | 0.003 |
| epoch | 25.18 | <0.0001 |
| groupXepoch | 0.381 | n.s. |
| (Wake) | F | P |
| --- | --- | --- |
| group | 11.43 | 0.001 |
| epoch | 15.00 | <0.0001 |
| groupXepoch | 1.91 | n.s. |
| (REM) | F | P |
| --- | --- | --- |
| group | 4.75 | 0.033 |
| epoch | 7.02 | 0.002 |
| groupXepoch | 0.24 | n.s. |
Supplemental Figure
